# Autosomal Dominant Polycystic Kidney Disease: From Pathophysiology of Cystogenesis to Advances in the Treatment

**DOI:** 10.3390/ijms23063317

**Published:** 2022-03-19

**Authors:** Jana Reiterová, Vladimír Tesař

**Affiliations:** 1 Department of Nephrology, First Faculty of Medicine, Charles University, General University Hospital in Prague, 128 08 Prague, Czech Republic; jana.reiterova@vfn.cz; 2 Institute of Biology and Medical Genetics, First Faculty of Medicine, Charles University, General University Hospital in Prague, 128 08 Prague, Czech Republic

**Keywords:** autosomal dominant polycystic kidney disease, cystogenesis, therapy

## Abstract

Autosomal dominant polycystic kidney disease (ADPKD) is the most common genetic renal disease, with an estimated prevalence between 1:1000 and 1:2500. It is mostly caused by mutations of the *PKD1* and *PKD2* genes encoding polycystin 1 (PC1) and polycystin 2 (PC2) that regulate cellular processes such as fluid transport, differentiation, proliferation, apoptosis and cell adhesion. Reduction of calcium ions and induction of cyclic adenosine monophosphate (sAMP) promote cyst enlargement by transepithelial fluid secretion and cell proliferation. Abnormal activation of MAPK/ERK pathway, dysregulated signaling of heterotrimeric G proteins, mTOR, phosphoinositide 3-kinase, AMPK, JAK/STAT activator of transcription and nuclear factor kB (NF-kB) are involved in cystogenesis. Another feature of cystic tissue is increased extracellular production and recruitment of inflammatory cells and abnormal connections among cells. Moreover, metabolic alterations in cystic cells including defective glucose metabolism, impaired beta-oxidation and abnormal mitochondrial activity were shown to be associated with cyst expansion. Although tolvaptan has been recently approved as a drug that slows ADPKD progression, some patients do not tolerate tolvaptan because of frequent aquaretic. The advances in the knowledge of multiple molecular pathways involved in cystogenesis led to the development of animal and cellular studies, followed by the development of several ongoing randomized controlled trials with promising drugs. Our review is aimed at pathophysiological mechanisms in cystogenesis in connection with the most promising drugs in animal and clinical studies.

## 1. Introduction

Autosomal dominant polycystic kidney disease (ADPKD) is the most common inherited disorder that typically presents in adults. Both kidneys are affected by cysts, leading to end stage renal disease in adulthood. Prevalence of ADPKD was estimated to be 1:400 to 1:1000 live births. Recent data from ERA registry presented lower prevalence of ADPKD ranging from 3.96/10,000 [1], but the study using measurement of frequency of high-confident mutations of ADPKD genes (*PKD1* and *PKD2*) in databases gnomAD and BRAVO estimated higher prevalence 9.3/10,000 [2]. The higher estimated prevalence detected in populations analyzed by DNA sequencing indicates that a significant proportion of affected patients with milder phenotype remains undiagnosed.

ADPKD is a progressive disease, leading to chronic kidney disease (CKD) and finally results in end-stage renal disease (ESRD). ESRD requiring renal replacement therapy (RRT) develops by the age of 70 years in up to 70% of patients with ADPKD [3]. ADPKD patients form about 10% of patients in dialysis and transplantation programs [4]. Cysts predominantly originate from distal tubules. Cysts gradually grow due to abnormal cell proliferation, fluid secretion and production of extracellular matrix. Glomerular filtration rate remains normal for years because of maladaptive glomerular filtration in the remaining nephrons. Total kidney volume evaluated by magnetic resonance reflects progressive cyst growth in patients. Some patients suffer from chronic pain because of cyst enlargement, acute pain can be caused by cyst infection or colic pain by nephrolithiasis. Arterial hypertension is present in 75% of patients with normal renal function from an early age. Cyst expansion is associated with intrarenal ischemia leading to the activation of renin-angiotensin-aldosterone system. On the other hand, angiotensin contributes to cyst expansion, increases oxidative stress, tubulointerstitial fibrosis, endothelin and sympathetic activity. Strict blood pressure management, particularly by blocking angiotensin, is the most important therapeutical option in ADPKD. Increased left ventricle mass was reported even in young normotensive ADPKD patients which is associated with higher cardiovascular mortality. Strict blood pressure control from childhood improves cardiovascular mortality of ADPKD patients.

ADPKD is a systemic disease because polycystins are expressed in many tissues. The systemic and multiorgan nature of ADPKD manifests by the occurrence of cysts in extrarenal organs such as the liver, pancreas and spleen, as well as by other affected organs such as cardiovascular abnormalities and brain vessel aneurysms. Polycystic liver disease is the most common extrarenal comorbidity accompanying ADPKD. Cyst volume of liver is frequently higher in females after multiple pregnancies and/or use of hormonal replacement therapy. The abnormality of connective tissues can be associated with hernias, diverticulosis and heart valve abnormalities in ADPKD patients.

In most cases, ADPKD is caused by mutations in 2 genes: *PKD1* (16p13.3) and *PKD2* (4q22.1). The germline mutations in PKD1 gene are present in about 80% of the ADPKD patients, mutations in PKD2 gene in the remaining 15% of ADPKD patients [5]. About 5% of patients probably harbor mutations in other loci.

## 2. Pathogenesis of ADPKD

### 2.1. Polycystins and Signaling Pathways

The gene products of *PKD1* and *PKD2* genes are polycystin-1 (PC1) and polycystin-2 (PC2). PC1 and PC2 modulate number of signaling pathways in cooperation with many other proteins. Subcellularly, both proteins can be found in plasma membrane and PC2 in endoplasmic reticulum membrane. The assembly of PC1/PC2 complex obtained by cryo-electron microscopy was first presented in 2018 [6]. PC1/PC2 complex was found to be formed by one PC1 and three PC2 molecules. This observation suggests that due to three positively charged, cavity-facing residues of PC1 protein, cation permeation of the channel may be inhibited.

**Figure 1:**
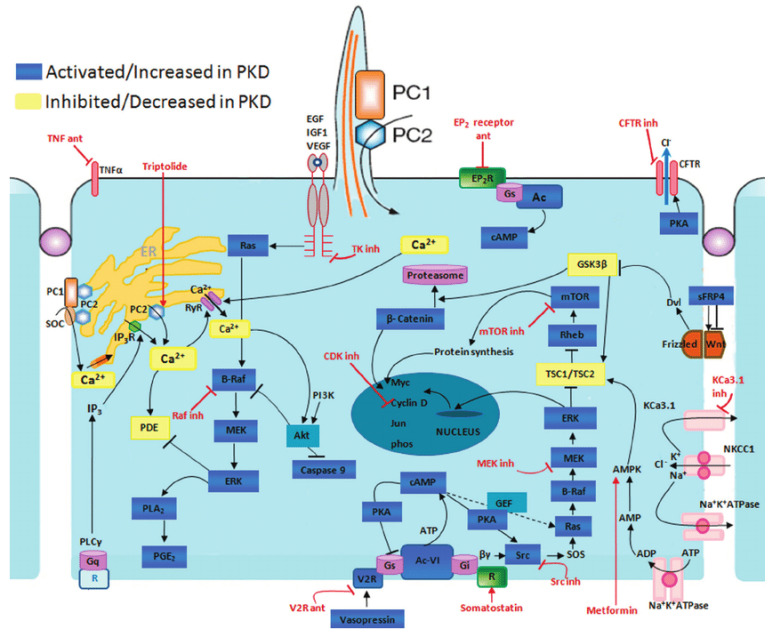
Disrupted pathways in polycystic kidney disease (PKD) and potential therapeutic intervention with specific agents. Agents associated with potential therapeutic interventions are represented in red; ant = antagonist; inh = inhibitor. AC-VI = adenylate cyclase 6; Akt = protein kinase B; AMPK = AMP kinase; B-Raf = v-raf murine sarcoma viral oncogene homolog B1; CDK = cyclin-de-pendent kinase; CFTR = cystic fibrosis transmembrane conductance regulator; EGF = epidermal growth factor; EP 2 R = E-prostanoid receptor 2; ERK = extracellular signal-regulated protein kinase; GSK3 = glycogen synthase kinase 3; IGF1 = insulin-like growth factor 1; IP 3 R = inositol 1,4,5-trisphosphate receptor; MAPK = mitogen-activated protein kinase; mTOR = mammalian target for rapamycin; PC1 = polycystin-1; PC2 = polycystin-2; PDE = phosphodiesterase; PGE 2 = prostaglandin E2; PI3K = phosphatidylinositol 3-kinase; PKA = protein kinase A; PLA 2 = phospholipase A2; PLCγ = phospholipase C-γ2; R = somatostatin sst2 receptor; Rheb = ras homolog enriched in brain; RyR = ryanodine receptor; sFRP4 = secreted Frizzled-related protein 4; TK = tyrosine kinase; TNF-α = tumor necro-sis factor-α; TSC = tuberous sclerosis proteins tuberin (TSC2) and hamartin (TSC1); V2R = vasopressin V2 receptor; VEGF = vascular endothelial growth factor [9].

The typical manifestation of ADPKD is the formation of renal cysts. Cysts are formed in about 1% to 3% of nephrons [7]. Kidney cysts are formed already prenatally but they are mostly detected in adults [8]. The cyst development and enlargement include numerous cellular changes. The resulting changes in polycystin expression cause the damage of several intracellular signaling pathways terminating in the development of cysts due to cell proliferation and fluid secretion (Figure 1).

### 2.2. Cyclic AMP Pathway

One of the typical features of cyst-lining cells is elevated cAMP levels. cAMP stimulates the growth of cystic cells through stimulation of protein kinase A and activation of the Ras/Raf/ERK pathway leading to proliferation and enlargement of cystic cells. Cell proliferation of extracellular matrix is also increased. Moreover, cAMP increases the fluid secretion (involving cystic fibrosis transmembrane conductance regulator-CFTR) causing cyst growth [10]. Abundance of inflammatory cells and abnormal cell-cell junctions are typical for cystic tissue [11]. The most important therapeutical option is the reduction of cAMP in cysts which is associated with the decrease of the cyst growth.

### 2.3. The TSC-mTOR Pathway

The negative effect of PC1 on TSC-mTOR pathway (tuberous sclerosis complex-mammalian target of rapamycin) was described in [12]. TSC complex consisting of proteins TSC1 (also called hamartin) and TSC2 (also called tuberin) plays a role as a negative regulator of mTOR kinase, as it acts as a GTPase-activating protein. mTOR complex regulates cell growth and proliferation, as well as actin cytoskeleton and apoptosis.

Carboxy-terminal tail (CTT) of PC1 directly interacts with tuberin and protects it from phosphorylation by Akt (protein kinase B). PC1 leads to retention of TSC2 at plasma membrane which allows TSC2 to remain bound to TSC1 in functional complex [13]. On the other hand, TSC2 is essential for PC1 localization at plasma membrane [14].

### 2.4. PI3K/Akt Pathway

C-terminus of PC2 interacts with IP3 receptor (IP3R) on endoplasmic reticulum and can prolong IP3-dependent calcium release [15]. On the contrary, PC1 has an ability to inhibit this calcium release as it weakens the interaction between PC2 and IP3R [16]. It was shown that in the cell with PC1 expression the PI3K/Akt (Phosphatidylinositol-4,5-bisphosphate 3-kinase/Protein kinase B) pathway is activated. In this pathway, activated receptors located at plasma membrane directly stimulate PI3K triggering conversion of phosphatidylinositol-3,4-bis-phosphate (PIP2) to phosphatidylinositol-3,4,5-tris-phosphate (PIP3). Protein kinase B (Akt) binds to PIP3 at the plasma membrane which allows PDK1 (3-phosphoinositide dependent protein kinase 1) to activate Akt by its phosphorylation [17].

Activated Akt can influence apoptosis, growth, proliferation, metabolism, angiogenesis, survival, protein synthesis and transcription [18]. Activation of PI3K/Akt pathway may decrease in level of intracellular calcium. PC2 is subsequently recruited to the plasma membrane where it forms complex with PC1 serving as an influx calcium channel [18]. PC1 induces the resistance to apoptosis by activation PI3K [18].

### 2.5. The JAK-STAT Pathway

Polycystins play a role in the cell cycle. Both polycystins modulate JAK-STAT pathway (The Janus kinase-signal transducers and activators of transcription). PC1 binds and activates JAK2 kinase that subsequently phosphorylates STAT proteins (STAT1 and 3). Phosphorylated STAT1 shifts to the nucleus to bind to p21 gene promoter which is associated with higher gene expression. Increased levels of p21 prevent cells from proliferating by arrest in G0 phase of cell cycle. PC2 is required as a cofactor of this activation [19].

STAT6 protein can be also activated by PC1 [20]. The C-terminal part of PC1 shifts to the nucleus and activates STAT6-dependent transcription. Cyst-lining cells with highly activated STAT6 have been described in animal models [21].

### 2.6. The Id Pathway

The Id pathway is associated with cell division. Id2 (Inhibitor of DNA binding 2) is a member of the helix-loop-helix family of proteins which bind to positively acting transcription factors and prevent them from binding to DNA. Putative binding partners for Id proteins were found to be transcription factors regulating cell such proteins as p21 [21]. PC2 can directly bind to Id2, Id2 is not able to bind E protein transcription. Moreover, the PC2-Id2 interaction is regulated by PC1-dependent serine phosphorylation of PC2 [22].

### 2.7. G-Protein Signaling Pathway

The cytosolic domain of PC1 activates transcription factor AP-1 (activating protein-1). AP-1 influences the cellular actions such as differentiation, proliferation and apoptosis [23]. Firstly, heterotrimeric G-proteins are activated by C-terminus of PC1. The activated Gα subunits activates c-Jun N-terminal kinase (JNK, member of MAPK kinases) that phosphorylates AP-1 transcription factor. In addition, JNK/AP-1 activation is supported by protein kinase C (PKC). AP-1 dependent transcription is triggered by PC2 interacting with PC1 [24].

Calcineurin/NFAT (nuclear factor of activated T-cells) signaling pathway regulates cell development and adaptation of different cell types [25]. PC1 activates trimeric G-protein that leads to Gα-dependent activation of phospholipase C (PLC) followed by release of calcium from endoplasmic reticulum. Increased Ca^2+^ concentration leads to activation of calcineurin, a Ca^2+^-dependent phosphatase. Dephosphorylated NFAT can enter into the nucleus and can influence the transcription of target genes [26].

Both families of AP-1 and NFAT transcription factors regulate to synergistically regulate gene expression of diverse genes with composite DNA elements containing adjacent NFAT and AP-1 binding sites [27]. This balanced activation is well described in the productive immune response but its possible effect on signaling pathways in other cells is yet to be proved.

### 2.8. Wnt Signaling Pathway

The Wnt signaling pathway regulates essential biological functions. Wnt proteins are growth factors. They play a roles in signaling pathways controlling proliferation, differentiation and cellular polarity during embryonal development [28]. It is divides into two major arms, the canonical Wnt/β-catenin pathway, and a β-catenin independent pathway that is responsible for establishing planar cell polarity and tissue morphogenesis.

In canonical Wnt pathway, secreted glycoproteins Wnt bind to Frizzled receptor (containing co-receptor LRP-low-density lipoprotein receptor-related protein). Activated Frizzled/LRP receptor can recruit cytoplasmatic protein Disheveled to the membrane providing a docking site for Axin and GSK-3 (glycogen synthase kinase 3) [29]. Axin targets β-catenin for ubiquitination. In case of activation of canonical Wnt pathway, β-catenin degradation is prevented. Therefore, the levels of β-catenin rise together with TCF (T cell factor) and promotes transcription of Wnt target genes [29]. Carboxy-terminal part of PC1 inhibits the interaction between β-catenin and TCF protein. The canonical Wnt pathway is activated in cystic tissue of ADPKD patients. PC1 could affect the cystogenesis during embryogenesis by the influence on Wnt pathway.

Noncanonical Wnt pathway plays role in determination of cellular polarity and motility and is required during tissue formation and homeostasis [30]. Activation of the noncanonical pathway leads to an increase of intracellular calcium. This pathway begins with the activation of the Frizzled receptor by Wnt ligands followed by G-protein-dependent activation of phospholipase C. Wnt can bind to the extracellular domain of PC1 which leads whole cell currents and Ca^2+^ influx dependent on PC2. Loss of cell polarity is frequently observed in cystic cells [31].

## 3. Treatments

### 3.1. Current Therapeutical Options

Healthy lifestyle and diet, maintenance of optimal weight, regular cardiovascular exercise and avoidance of smoking are generally recommended in ADPKD. High water intake is used as a prevention of nephrolithiasis but also leads to the suppression of vasopressin which could lead to an increase of fluid secretion into cysts. However, the results of trials with increased water intake were not conclusive. Salt intake should not exceed 5 g per day as all patients with other renal diseases.

Strict control of hypertension is the most important therapeutical option. Hypertension often presents in children and young adults with ADPKD and later promotes decline of renal function. Especially left ventricle hypertrophy was very common before greater attention was paid to treating blood pressure appropriately. RAS blockade is the mainstay of treatment of hypertension in ADPKD. The goal blood pressure should be less than 110/75 mm Hg in younger people than 50 years with eGFR above 60 mL/min per 1.73 m^2^. ACEI are recommended as the first safe choice [32]. The HALT study confirmed that the strict control of blood pressure decreases the mass of left ventricle, renal vascular resistance, proteinuria and kidney volume. Unfortunately, there was no significant effect of lower blood pressure on the slope of eGFR. The combination of ACEI and AT1 blockers was found to be safe even in ADPKD with advanced renal insufficiency but not more effective than monotherapy [33].

### 3.2. Tolvaptan

Tolvaptan is a selective arginine vasopressin type 2 receptor antagonist. Tolvaptan reduced the progression of kidney function decline in ADPKD in animal models [34,35]. The role of arginine vasopressin-mediated cAMP as a driver of fluid secretion into the cysts in ADPKD was demonstrated in preclinical studies. Two multicentric, randomized, placebo-controlled trials evaluated the safety and efficacy of tolvaptan in ADPDK patients. The TEMPO 3:4 trial (NCT00428948, ClinicalTrials.gov) enrolled 1445 ADPKD patients with normal renal function or mild renal insufficiency (CKD1, CKD, baseline eGFR ≥ 60 mL/min per 1.73 m^2^). Tolvaptan reduced an increase in kidney volume a 2.8% per compared with a 5.5% per year increase in the placebo group (*p* ˂ 0.001) over the 3-year study period. Tolvaptan also slowed a decline in kidney function, as measured by reciprocal of serum creatinine level, compared with placebo, −2.61 vs. −3.81 per year (*p* ˂ 0.001) [36]. As expected, there were adverse aquaretic events, such as thirst, polyuria, nycturia, polakisuria and polydipsia. Moreover, clinically important liver involvement with increase of liver enzymes was described in 4.4% of patients. Liver enzymes in all patients normalized after cessation of tolvaptan. TEMPO 3:4 was followed by open label study TEMPO 4:4, the decrease of eGFR decline was found in the following two years [37]. A second trial, REPRISE (NCT02160145) was designed to evaluate the efficacy and safety of tolvaptan [38]. A one-year study in ADPKD patients with more advanced disease (eGFR 25–65 mL/min per 1.73 m^2^) showed a reduction in decline of eGFR in treated patients. The post hoc analysis from open label extension found that tolvaptan also delayed eGFR decline in CKD4 (with eGFR 15 to 24 mL/min per 1.73 m^2^) [39]. A monthly protocol for monitoring liver enzymes is obligatory for 18 months as a prevention of severe hepatic toxicity, followed by 3-month check-up.

Nowadays, therapy with tolvaptan should be recommended to all ADPKD patients with probable rapid progression of the disease. Criteria for tolvaptan use are proposed as follows: 1. age 18–55 years, 2. chronic kidney disease CKD1–4 (eGFR ≥ 25 mL/min per 1.73 m^2^), 3. high risk measured by available risk scores (longitudinal diameter > 17 cm by ultrasound, total kidney volume > 750 mL, Mayo imaging classification 1C, 1D, 1E or PROPKD score > 6), and 4. rapid decline of eGFR 3 mL/min per > 1.73 m^2^) for 5 years. Mayo imaging classification is based on magnetic resonance and clearly predicts decline in eGFR on the basis of initial total kidney volume [40]. PROPKD (Predicting Renal Outcomes in Polycystic Kidney Disease) score predicts the prognosis according to molecular genetic analysis in combination with simple clinical information (sex, age at diagnosis of hypertension, age of first episode of macroscopic hematuria, flank pain related to cysts) [41]. Risk stratification is important because there was no difference of eGFR decline in patients having low risk of ADPKD progression [42]. Tolvaptan is not recommended in ADPKD patients with predicted mild clinical course.

### 3.3. Somatostatin Analogues

Somatostatin binds to five G protein-coupled receptors and inhibits directly and indirectly intracellular cAMP production in liver and kidney. This inhibition results in inhibition of fluid secretion, cell proliferation and induction of apoptosis. Somatostatin is rapidly eliminated and therefore analogues such as octreotide, lanreotide and pasireotide with a longer half-life have been synthesized. The efficacy of somastatin analogues on total kidney volume and on the decrease of eGFR was demonstrated in phase 2 small studies [43,44].

Octreotide did not significantly slow down the decline in eGFR and the increase of polycystic kidney volume after 3 years in a phase 3 trial ALADIN [45]. Somatostatin analogues led to decrease in GFR during first three months because of a decline in maladaptive hyperfiltration. Later, the effect is followed by a slower decline of eGFR because of the structural beneficial effect. Trials with such drugs require longer duration. A post hoc analysis of ALADIN study confirmed that octreotide had a beneficial effect on the slope of measured GFR from the first to the third year of treatment.

A larger, open-label randomized controlled trial (DIPAK 1 study) was performed in the Netherlands, investigating the effects of lanreotide in 305 ADPKD patients with CKD3 after 2.5 years of treatment [46]. Lanreotide significantly reduced the growth of liver and kidney cysts. There was found no influence on eGFR. Nowadays, there are three studies with somatostatin ongoing or finalized.

On the other hand, promising data exists for somatostatin analogues for treatment of polycystic liver disease. In an open label, 6-month placebo-controlled trial of 27 patients treated with 120 mg of lanreotide versus 27 on placebo, lanreotide led to a 2.94% decrease of total liver volume compared with a 1.6% increase in placebo group [47]. In extension phase, the decrease of liver volume remained, but there was increase by 4% after cessation of treatment. Similarly, therapy with octreotide in an American study led to 4.7% decrease of liver volume [48]. Quality of life was also improved. Unfortunately, side-effects such as diarrhea, nausea, abdominal pain, meteorism, cholelithiasis and hyperglycemia can limit the use of somatostatin analogues. Higher prevalence of hepatic cyst infection was also reported.

Nowadays, there is no evidence that somatostatin analogues should be used in patients with polycystic renal disease. However, they should come into account in patients with high volume polycystic liver to put off liver transplantation.

## 4. Future Options

### 4.1. Targeting the cAMP Pathway

#### 4.1.1. Lixivaptan

Lixivaptan is a selective V2 receptor antagonist. There was found more than 50% reduction in cysts volume in the kidney of polycystic rats [49]. A 23% reduction in cAMP levels was shown. Lixivaptan has a lower risk of hepatotoxicity than tolvaptan [50]. The study with 1200 ADPKD patients, with CKD stages 1–3, is ongoing (http://www.clinicaltrial.gov:NCT04064346 (accessed on 1 February 2022)).

#### 4.1.2. Cystic Fibrosis Transmembrane Conductance Regulator (CFTR) and Potassium Channel Inhibitors

CFTR is a chloride ion channel which is located in the apical membrane of the epithelial cells of the liver, and proximal and distal tubules of the kidney. Transtubular secretion to the cysts is facilitated by chloride secretion through the CFTR ion channel that acts as an electrochemical driving force. Milder clinical course was described in 3 ADPKD patients with cystic fibrosis with mutation of gene encoding CFTR channel, as compared with siblings with ADPKD without cystic fibrosis [51].

Lumacaftor (VX-809 corrector of CFTR) interacts with CFTR protein in the membrane and reduced cyst growth in vitro and in mouse model [52]. The cyst phenotype changes from fluid secretion to fluid reabsorption. A randomized, double-blind, placebo-controlled multicentric study evaluating the efficacy, safety, tolerability and pharmacokinetics of the orally administered CFTR corrector GLPG2737 in ADPKD patients has been recently initiated. (Galapagos, EudraCT2019-003521-21).

Steviol glycosides, which are sweet but do not induce a glycemic response after resorption, inhibit cAMP-activated chloride secretion by targeting CFTR in high doses. It inhibited cyst growth in cell culture by CFTR degradation in proteasome [53]. The proton pump inhibitor lansoprazole also reduced the cyst growth in vitro and in PCK rats by down-regulation of CFTR [54].

TRAM-34—the clotrimazole analogue-inhibits specifically K^+^ channel KCa3.1 and leads to the inhibition of Cl^-^ secretion without affecting CFTR channel. It inhibits cyst formation and it was found to be safe in patients with sickle cell disease [55].

#### 4.1.3. TMEM16A Inhibitors

Chloride channel TMEM16A activated by calcium ions was found to be important for fluid secretion into renal cysts in vitro [56]. TMEM16A-inhibitors decreased the activity of chloride channel. Niclosamide and benzbromarone inhibited TMEM16A and the treatment with these drugs was associated with decrease of cyst growth [57]. TMEM16A specific small molecule Ani9 also led to the inhibition of cystogenesis in vivo [58]. TMEM16A-inhibitors not only block chloride currents but inhibit expression TMEM16A during long treatment. TMEM16A-inhibitors seem to be promising for ADPKD in humans in near future.

### 4.2. Targeting the EGF Receptor Pathway

#### 4.2.1. Bosutinib

Increased Src activation has been recently described in animal models with polycystic kidney disease. Bosutinib, Src/Bcr-Abl tyrosin kinase inhibitor, decreased cyst proliferation, adhesion and migration in PKD mouse and rat models [59]. Src inhibition correlates with decreased EGFR (ErbB1) and ErbB2 activity. Src also inhibits the B-Raf/MEK/ERK signaling pathway without reducing elevated cAMP.

Bosutinib was shown to reduce the renal growth rate evaluated by MR in a phase 2 study [60]. The influence on eGFR decline was not statistically significant at the end of 3 years of follow up. The adverse effects, such as diarrhea, nausea and elevated liver enzymes, were significantly more frequent in the treatment group than in the placebo group.

#### 4.2.2. Tesevatinib

Tesevatinib is a unique multi-kinase inhibitor that is being used in a phase II clinical trial for ADPKD treatment (http://www.clinicaltrial.gov:NCT01559363 (accessed on 1 February 2022)). In mouse model of autosomal recessive polycystic kidney disease, it inhibited spectrum of key tyrosine kinases (EGFR, HER2/ErbB2, c-Src) that are associated with cell proliferation and angiogenesis. Inhibition of cSrc profile causes the decreased activity of both EGFR axis and cAMP pathways in cysts [61].

#### 4.2.3. Thiazolidinediones

Thiazolidinediones are used to treat type 2 diabetes mellitus. These drugs reduce proliferation, fibrosis and inflammation through inhibiting transforming growth factor beta [62]. Pioglitazone also reduces CFTR gene expression in vitro models [63]. High-dose pioglitazone improved renal survival in Pkd1 negative mouse models. In a PKD mouse model, combination of pioglitazon and tolvaptan reduced renal polycystosis more than pioglitazon alone [64]. A phase 2 clinical trial with low-dose pioglitazon in ADPKD patient has been recently finished (http://www.clinicaltrial.gov:NCT02697617 (accessed on 1 February 2022)).

### 4.3. Targeting AMP-Activator Protein Kinase

#### 4.3.1. Metformin

Metformin is a biguanide drug, which is used in type 2 diabetes, Metformin activates 5′AMP-activated protein kinase (AMPK) that inhibits CFTR by phosphorylation which leads to the suppression of epithelial fluid and electrolyte secretion [65]. Furthermore, AMPK phosphorylates tuberin protein and leads to indirect inhibition of the mTOR pathway. Moreover, fatty fat oxidation is also impaired in polycystic kidney disease, which metformin can positively influence [66]. Metformin is a promising drug in the treatment of early stages of ADPKD according to observational studies. Metformin slowed cystogenesis in a Zebrafish model with deficient polystin-2 [67]. On the other hand, metformin is not safe in patients with advanced renal failure, where it can cause lactic acidosis. The results of a 2 year randomized study (TAME PKD) have been recently published [68]. Metformin was found to be a safe drug in ADPKD non-diabetic patients, but there was only non-significant trend to lower eGFR loss in metformin treated subjects. Another study, METROPOLIS, will enroll 150 non-diabetic patients ranging from 18 to 50 years of age, with eGFR ≥ 45 mL/min/1.73 m^2^ and truncating PKD1 gene mutation. Patients will be randomized to metformin or tolvaptan and after 25 months the assessement of TKV and eGFR will be done (http://www.clinicaltrial.gov:NCT03764605 (accessed on 1 February 2022)).

#### 4.3.2. Statins

Statins are recommended in all patients with reduced glomerular filtration rate. Statins probably activate AMPK and the amelioration of cystic proliferation was observed in ADPKD animal models [69]. A clinical trial of 110 ADPKD children and young adults showed the beneficial effect of pravastatin on total kidney volume [70]. The use of statins could already come into account in young ADPKD patients with normal kidney function. On the other hand, no significant effect of statins was found in a post hoc analysis of the HALT-PKD trial [71]. Recent recruiting interventional studies are summarized in Table 1.

### 4.4. Targeting MAPK Pathway-Raf Kinase Inhibitors

Raf kinases are part of MAPK cascade where ERK is finally phosphorylated and shifted to the nucleus regulating different transcription factors [72]. Sorafenib is a multikinase inhibitor used for the treatment of advanced renal cell carcinoma. Sorafenib in low concentration inhibited cyst growth by Raf-inhibition in vitro. PLX5568 selectively inhibits a Raf kinase and slowed the cyst growth in a rat ADPKD model, but the renal function did not improve because of increased renal fibrosis [73].

### 4.5. Dietary Interventions in ADPKD

Polycystic kidneys are under high metabolic demand, the growth of cyst epithelial cells is highly dependent on glucose consumption. There is a shift in energy production from mitochondrial oxidative phosphorylation to aerobic glycolysis in cyst cells (Warburg effect). The pentose phosphate pathway and fatty acid biosynthesis are abundant. On the other hand, fatty acid oxidation and oxidative phosphorylation are diminished. Polycystins have been recently shown to regulate directly mitochondrial function which could be associated with various metabolic abnormalities in ADPKD [74].

#### 4.5.1. Caloric Restriction

Moderate food restriction slowed cyst growth through AMPK activation and mTOR signaling suppression in rodent models [75]. Dietary strategies include daily caloric restriction, intermittent fasting, and a ketogenic diet. Decrease of 30–50% of caloric intake decrease the cyst growth in animal models without signs of malnutrition [76,77].

Obesity is a strong independent predictor of ADPKD progression according to HALT study [78]. Caloric restriction, intermittent fasting, time-restricted feeding, a ketogenic diet and 2-deoxy-glucose may improve the prognosis of ADPKD. Preclinical studies targeting glucose metabolism by intermittent fasting are promising. The evidence for salt restriction is growing [79]. The results of long-term randomized human intervention studies based on dietary interventions in ADPKD are not yet available (Table 2).

#### 4.5.2. 2-Deoxyglucose (2DG)

Metabolic derangement in ADPKD have been recently described. Increased aerobic glycolysis dominates in cystic cells. 2DG functions as a competitive inhibitor of the glycolytic pathway after a transport into cystic cells. Chronic administration of low dose of 2DG prevented the progression of cyst growth and was safe in animal models [80]. 2DG is safe and efficient drug in animal models. This molecule seems to be a promising drug in a clinical trial.

#### 4.5.3. mTOR Inhibitors

Mutations in PKD genes lead to activation of rapamycin kinase complex. mTOR inhibitors inhibited the Warburg effect. Moreover, mTOR inhibitors downregulate cyclin A, B, 1D and E, which are associated with cell cycle and lead to abnormal proliferation of cystic cells. In spite of the success in preclinical animal studies, clinical trials with sirolimus and everolimus did not show a positive effect on renal prognosis in human ADPKD [81,82]. A hypothesis proposed that the dosing of mTOR inhibitors was insufficient because of risk of nephrotoxicity. Positive preclinical animal studies do not often translate into positive clinical human studies.

### 4.6. Targeting the KEAP1-Nrf2 Pathway

Bardoxolone activates a Nrf2 pathway that improves renal function in patients with diabetes mellitus 2 (BEAM trial) [83]. A further trial (BEACON trial) had to be stopped for more frequent heart failure in the bardoxolone group [84]. The absence of Nrf2 accelerated cystogenesis in the ADPKD model, whereas an induction of Nrf2 slowed cystogenesis [85]. The Falcon study in a phase 3 with bardoxolone in 300 ADPKD patients is now ongoing (http://www.clinicaltrial.gov:NCT03918447, accessed on 1 February 2022)).

### 4.7. Targeting Intracellular Calcium Regulation and Cell Cycle Regulation

#### 4.7.1. Calcimimetics

Calcium-sensing receptor activation by calcimimetics leads to decrease of cAMP and increase of intracellular calcium. Calcimimetic R568 significantly reduced the volume of cysts in the kidneys of mice [86]. In the ADPKD rats and mice, the combined treatment with lixivaptan and calcimimetic R568 reduced kidney weight, cyst and fibrosis volume more effectively than the individual drug [87].

#### 4.7.2. Triptolide

Triptolide was from Tripterygium wildfordii in China. It was found to have antiproliferative and antiapoptotic properties. Triptolide induced a release of calcium from the endoplasmatic reticulum. It is dependent on PC2. The elevated calcium was associated with decrease of cyst growth in a mouse cystic model. The cyclin p21 expression was proved. Moreover, triptolide inhibited cell cycle in the smooth muscle cells after induction by platelet-derived growth factor (PDGF) [88]. Triptolide also inhibited cell proliferation in renal cell carcinoma. Triptolide led to cell cycle arrest in G0 phase, which supports the role triptolide in ADPKD model.

There were only published the results of uncontrolled trial with 9 proteinuric ADPKD patients. Triptolide led to decrease of proteinuria but the volume of polycystic kidney and eGFR were not influenced [89].

#### 4.7.3. Cyclin Dependent Kinases (CDK) Inhibitors

PC1 inhibits cell cycle by cyclin-dependent kinase 2 through up-regulation of p21. PC2 binds to protein, which regulates cell proliferation and differentiation. PC2-protein complex is not able to translocate into the nucleus. Roscovitine, CDK inhibitor, reduced cystic kidney and liver progression in mouse ADPKD models [90].

#### 4.7.4. Histon Deacetylase 6 Inhibitors (HDACi)

HDAC6i significantly decreased a release of calcium ions from endoplasmatic reticulum through PC2. Moreover, HDAC6i may influence the acetylation of α-tubulin which is important in cell polarity and/or microtubule assembly [91]. Valproic acid decreased cyst growth in Pkd2-deficient mice [92].

### 4.8. Targeting Interstitial Changes

#### 4.8.1. Venglustat

Activation of rapamycin kinase complex lead to de novo ceramide synthesis and to an increase of glucosylceramide production. Accumulation of glycosphingolipids leads to the loss of cell differentiation and proliferation which promotes cyst growth [93]. Glucosoceramide plasma levels were found in animal models and treatment with glucosylceramide synthase inhibitors reduced cyst growth [94]. STAGED-PKD trial with venglustat recruited ADPKD patient with progressive disease since 2018. The trial was finished earlier in June 2021, because no effect on the volume of polycystic kidney and on the decrease of eGFR was found.

#### 4.8.2. Inflammation Inhibitors

Interstitial inflammation in cystic kidney contributes to the decrease of renal function. Macrophages are the principle cells of the inflammatory interstitium in PKD animal models. Bindarit is an inhibitor of a monocyte chemoattractant protein-1 (MCP-1) and improved the prognosis of polycystic rats [95]. Etanercept, a tumor necrosis factor alpha inhibitor, used in rheumatology, decrease the growth of cysts [96].

TWEAK is a TNF superfamily cytokine is associated with increased response to inflammation and cell proliferation. Anti-TWEAK antibodies significantly decreased cyst growth in polycystic mice [97].

### 4.9. MicroRNAs Blockers

MicroRNAs are short noncoding RNAs that bind to target mRNAs inhibiting the translation.

Some miRNAs are upregulated in progressive ADPKD. Anti-miRNA novel drugs may probably improve the growth of cysts. Two microRNAs, 17 a 21, are both upregulated in kidney cysts [98]. MicroRNA-17 enhances cyst proliferation and microRNA-21 inhibits apoptosis.

RGLS4326, short oligonucleotide inhibitor of microRNA-17 (miR-17 is nowadays studied. RGLS4326 was found to be safe and efficient in ADPKD animal models after subcutaneous administration [99]. On the other hand, microRNA-192 and -194 were down-regulated with hypermethylation in late stages of cystogenesis, suggesting their potential impact on cyst expansion. The therapeutic effect of microRNA-192 and -194 in vivo and in vitro were demonstrated. Injections of miRNAs precursors decrease the rate of cyst growth in Pkd1 knockout mice [100]. miR-192 and -194 seems to be a promising and safe therapy of ADPKD.

There are three interventional studies which start recruiting ADPKD patients early. Metformin as an inexpensive and familiar drug will be involved it two studies. Furthermore, the medication that could slow the frequent urination related to tolvaptan will be studied (Table 3).

## 5. Conclusions

Exciting advances have been made regarding the diagnosis, prognosis and therapy of ADPKD. Pathophysiological mechanisms on molecular are intensively studied in ADPKD. Many preclinical models have provided new therapeutic targets, but they do not have clearly to predict the clinical efficacy in humans. A disease modifying therapeutic drug (tolvaptan) is nowadays available. Encouraging results are expected from ongoing clinical trial testing promising molecules, such as lixivaptan, bardoxolone, pravastatin, pioglitazon, tesevatinib and CFTR corrector. Clinical research plays a most important role in understanding to the potential therapeutic efficacy of promising molecules. The findings of the trials will help clinicians to modify the natural history of ADPKD.

## Figures and Tables

**Table 1:** Recruiting interventional studies in ADPKD.

Study Title	Intervention	ADPKD Patients	Duration
Statin therapy in patients with early stage ADPKD (NCT03273413)	pravastatin 40 mg	200	2 years
A trial of bardoxolone methyl in patients with ADPKD (FALCON) (NCT03918447)	bardoxolone mehyl placebo	550	52 weeks
An extended access program for bardoxolone methyl in patients with CKD (EAGLE) (NCT03749447)	bardoxolone methyl (open label)	480	5 years
To evaluate the safety, tolerability, pharmacokinetics and pharmacodynamics of oral ALO1211 in healhy volunteers and in ADPKD subjects (NCT04908462)	AL01211 placebo	98	6 months
Efficacy and safety of Lixivaptan in the treatment of ADPKD (NCT04064346)	Lixivaptan placebo	1200	2 years
Safety of lixivaptan in subjects previously treated with tolvaptan fo ADPKD (ALERT) (NCT04152837)	lixivaptan (open label)	50	52 weeks

**Table 2:** Dietary studies in ADPKD.

Study	Title ADPKD Patients	Duration
Ketogenic dietary interventions in ADPKD (Keto-ADPKD) (NCT04680780)	63	3 months
Daily caloric restriction in ADPKD (NCT04907799)	126	2 years
Dietary intervention in ADPKD on tolvaptan (NCT03858439)	all on tolvaptan form Hamilton Nephrology clinic (Canada)	3 months
Time restricted feeding in ADPKD (NCT04534985)	30	2 years

**Table 3:** Starting studies.

Study Title	Interventions	ADPKD Patients	Duration
Metformin vs. Tolvaptan for treatment of ADPKD (NCT03764605)	drug: metformin drug: tolvaptan	150 non-diabetic with PKD1 truncating mutation	25 months
Implementation of Metformin herapy to ease decline of kidney function in polycystic kidney disease (IMPEDE-PKD) (NCT04939935)	drug:metformin drug:placebo	1164	104 weeks
PB to treat hereditary nephrogenic diabetes insipidus, ADPKD treated with tolvaptan, and severely polyuric patients with previous lithium administration (SerependityPB1) (NCT05190744)	drug: PB	20 on tolvaptan	1 year
